# Blockade of Piezo2 Pathway Attenuates Inflammatory and Neuropathic Pain in the Orofacial Area

**DOI:** 10.1155/2024/9179928

**Published:** 2024-09-27

**Authors:** Min-Jeong Jo, Jo-Young Son, Yu-Mi Kim, Jin-Sook Ju, Min-Kyoung Park, Min-Kyung Lee, Dong-Kuk Ahn

**Affiliations:** ^1^ Department of Oral Physiology School of Dentistry Kyungpook National University, Daegu, Republic of Korea; ^2^ Department of Dental Hygiene Kyung-Woon University, Gumi, Republic of Korea; ^3^ Department of Dental Hygiene Dong-Eui University, Busan, Republic of Korea

**Keywords:** acute pain, antinociception, interleukin-1*β*, neuropathic pain, Piezo2

## Abstract

Although previous studies suggest that Piezo2 regulates chronic pain in the orofacial area, few studies have reported the direct evidence of Piezo2's involvement in inflammatory and neuropathic pain in the orofacial region. In this study, we used male Sprague Dawley rats to investigate the role of the Piezo2 pathway in the development of inflammatory and neuropathic pain. The present study used interleukin (IL)-1*β*–induced pronociception as an inflammatory pain model. Subcutaneous injection of IL-1*β* produced significant mechanical allodynia and thermal hyperalgesia. Subcutaneous injection of a Piezo2 inhibitor significantly blocked mechanical allodynia and thermal hyperalgesia induced by subcutaneously injected IL-1*β*. Furthermore, the present study also used a neuropathic pain model caused by the misplacement of a dental implant, leading to notable mechanical allodynia as a consequence of inferior alveolar nerve injury. Western blot analysis revealed increased levels of Piezo2 in the trigeminal ganglion and the trigeminal subnucleus caudalis after inferior alveolar nerve injury. Furthermore, subcutaneous and intracisternal injections of a Piezo2 inhibitor blocked neuropathic mechanical allodynia. These results suggest that the Piezo2 pathway plays a critical role in the development of inflammatory and neuropathic pain in the orofacial area. Therefore, blocking the Piezo2 pathway could be the foundation for developing new therapeutic strategies to treat orofacial pain conditions.

## 1. Introduction

Pain is a physiological phenomenon designed to signal unpleasant sensory and emotional experiences associated with actual or potential tissue damage [1]. Chronic pain, including neuropathic pain, is one of the most problematic comorbidities that impairs and interferes with quality of life. It affects physical, emotional, and cognitive functioning due to its persistent nature over a long period of time [2–4]. Chronic pain can be caused by many different factors, and diseases can also be the underlying cause of chronic pain. Inflammatory pain occurs when chemical mediators responsible for tissue inflammation act on nociceptive nerve endings to lower the threshold for stimulation or sensitize the afferent firing rate. This process leads to allodynia and hyperalgesia, which are increased sensitivity to pain and heightened pain response, respectively [5]. Neuropathic pain, especially stemming from neurological lesions, is very challenging to treat due to the involvement of a variety of causes and mechanisms [2, 6]. Characterizations of neuropathic pain show persistent, paroxysmal symptoms associated with paresthesia or allodynia. However, existing painkillers, such as nonsteroidal anti-inflammatory drugs (NSAIDs), anticonvulsants, and opioids, are either ineffective in treating neuropathic pain or are accompanied by side effects [2].

Piezo is an ion channel that is activated by mechanical stimulation, such as pressure and shear stress, in various tissues and organs [7–9]. There are two types of Piezo channels, namely, Piezo1 and Piezo2, which consist of nonselective cation channels [10, 11]. Piezo1, mainly expressed in nonsensory tissues, is a mechanotransducer that initiates calcium ion signaling [12–14]. On the other hand, Piezo2 is mainly expressed in sensory neurons and plays an essential role in sensory processes such as tactile sensation and mechanical nociception [13]. Furthermore, Piezo2 plays crucial roles in light touch and proprioception both in mice and in humans [15].

Several studies have provided supporting evidence for the involvement of Piezo2 in pain information processing. Piezo2 is expressed in small myelinated (A*δ*) axons of sensory neurons [16], and Piezo2 knockout mice did not show allodynia following nerve injury or capsaicin-induced inflammation [17, 18]. Moreover, in *in vitro s*tudy, the application of D-GsMTx4, a Piezo2 inhibitor, blocked mechanosensitive current in enterochromaffin (EC) cells [19]. Recent studies have also demonstrated that the Piezo2 pathway participates in pain transmission in the orofacial region. Piezo2 expression was observed in all types of axons but predominantly in A*δ* axons in the sensory roots of the trigeminal ganglion [16]. Moreover, infraorbital nerve injury increased Piezo2 expression in the trigeminal ganglion [20]. Although these results suggest that Piezo2 is involved in chronic pain in the orofacial region, few studies have reported direct evidence of the role of the Piezo2 pathway in inflammatory and neuropathic pain in the orofacial region.

Here, we investigated the role of Piezo2 as a potential modulator in chronic pain, specifically focusing on its involvement in inflammatory and neuropathic pain in the orofacial region. We used interleukin (IL)-1*β*–induced pronociception as an inflammatory pain model. The effects of blocking the Piezo2 pathway on IL-1*β–*induced pronociception were examined following the subcutaneous injection of the Piezo2 inhibitor. The present study also assessed mechanical allodynia induced by inferior alveolar nerve injury as a neuropathic pain model. Changes in Piezo2 expression in the trigeminal ganglion and trigeminal subnucleus caudalis were investigated in experimental animals with inferior alveolar nerve injury. In addition, changes in neuropathic mechanical allodynia were examined after subcutaneous and intracisternal injection of a Piezo2 inhibitor.

## 2. Materials and Methods

### 2.1. Animals

Total 137 adult male Sprague Dawley rats weighing approximately 220–270 g were used in the present experiments. All animals were housed in an environment maintained at 23 ± 1°C with a 12-h light–dark cycle. Feed and water were provided ad libitum. All experimental procedures involving animals were approved by the Kyungpook National University Laboratory Animal Care Committee (approval code: KNU 2023-0050). In addition, all animal experiments adhered to the ethical guidelines of the International Association for the Study of Pain (IASP) established for the study of experimental pain in conscious animals. All animals were acclimatized to the experimental environment for at least 30 min before the start of the experiment, and each rat was used only once. All experiments were designed using a blinded approach.

### 2.2. Animals Models for Orofacial Pain

#### 2.2.1. IL-1*β*–Induced Pronociception

Mechanical allodynia and thermal hyperalgesia were examined following the subcutaneous injection of IL-1*β* into the vibrissa pad. In a previous study, the administration of 1 ng IL-1*β* resulted in significant mechanical allodynia and thermal hyperalgesia within 30 min, and IL-1*β*–induced pronociception persisted for up to 24 h [21]. The present study utilized subcutaneously injected IL-1*β* to induce pain behavior as an inflammatory pain model.

#### 2.2.2. Trigeminal Neuropathic Pain

The experimental rats were anesthetized with a mixed solution of ketamine (40 mg/kg) and xylazine (4 mg/kg). After anesthesia, the mandibular left second molar was extracted, and a mini dental implant (1 mm in diameter and 4 mm in length, MegaGen Donation, Gyeongsan, Korea) was implanted to intentionally damage the inferior alveolar nerve as previously described [22, 23]. Only data from rats that experienced inferior alveolar nerve damage due to improperly positioned dental implants were included in the final analysis.

### 2.3. Intracisternal Catheterization

For intracisternal injection, each rat was fixed on a stereotaxic frame, and a polyethylene tube 10 (PE-10) was inserted into the cisterna magna of anesthetized rats, as previously described [24–27]. In brief, a small hole was made in the atlantooccipital membrane and dura using a 27-gauge needle. PE-10 was then inserted intracisternally through this hole, with the cannula tip oriented toward the dorsal side of the obex. After being placed subcutaneously on the skull, PE-10 was secured using dental acrylic resin and stainless screws. Animals were allowed to recover for 5 days after surgery, a period previously proven to be sufficient [28, 29]. After inserting PE-10 into the intracisternal space, data from animals that showed blockage or malposition of PE-10 were excluded.

### 2.4. Assessment of Mechanical Allodynia

In the measurement of mechanical allodynia, previously described methods were used [30, 31]. For animal behavior analysis, each animal was placed in a custom-made plastic bin in a noise-free, dark room. Animals were acclimatized to the experimental environment for at least 30 min before behavioral evaluation. Behaviors such as aggressive biting and avoidance responses to air-puff stimulation were assessed. Application of air-puff stimulation was used as previously described and was performed 10 times continuously for 4 s at 10-s intervals [30, 32, 33]. The intensity and spacing of air-puff pressure were controlled using a Pico-Injector (Harvard Apparatus, Holliston, MA, USA). Air-puff stimulation was applied 1 cm away at a 90° angle to the skin at the site of IL-1*β* injection using a 10-cm-long 26-gauge metal tube. Mechanical allodynia was assessed when experimental animals exhibited nociceptive behavioral responses to 50% of air-puff stimuli. The cutoff pressure was 40 psi, as previously described [34–36]. Naive rats did not show withdrawal responses to pressures below 40 psi.

### 2.5. Assessment of Thermal Hyperalgesia

After applying radiant heat, the head withdrawal latency was determined as described previously [36, 37]. In this study, an infrared thermal stimulator (Infrared Diode Laser, LVI-808-10; LVI Tech, Seoul, Korea) was used to apply thermal stimulation to the site where IL-1*β* was injected, located on the vibrissa pad 10 cm away from the heat source. The intensity of the thermal stimulus was maintained with a stable latency of approximately 10–12 s. Each rat received two stimulations, with the interval between stimulations being at least 5 min for each trial. The blocking time was set at 20 s to prevent potential tissue damage.

### 2.6. Western Blot Analysis

Western blotting analyses were performed on samples from individual animals. On postoperative Day (POD) 5, naïve, sham, and trigeminal neuropathic pain rats were sacrificed under anesthesia, and the ipsilateral trigeminal ganglion and trigeminal subnucleus caudalis were excised and promptly frozen in liquid nitrogen. The samples were homogenized in extraction buffer containing 25 mM bicine, 150 nM sodium chloride (pH 7.6), 0.5 M EDTA, phosphatase inhibitor, and protease inhibitor. After extraction, the homogenates were centrifuged at 13,000 rpm for 30 min and the protein concentration of the supernatant was determined using the Qubit protein assay (Thermo Fisher, Waltham, MA, USA). Samples containing 20 (trigeminal ganglion) or 30 *μ*g (trigeminal subnucleus caudalis) were electrophoresed using NuPAGE 3%–8% Tris-acetate gel (Invitrogen, Waltham, MA, USA), followed by transfer onto nitrocellulose membranes. Protein transfer was verified by Ponceau S staining, and the membrane was blocked with 5% nonfat milk for 1 h at room temperature. Membranes were subsequently incubated overnight at 4°C with an anti-Piezo2 antibody (1:2000; NOVUS, MN, USA) and then with a secondary anti-rabbit IgG antibody (1:5000; Bio-Rad, Hercules, CA, USA). Finally, the band signal was visualized using the enhanced chemiluminescence (ECL) kit (Millipore Imager 600; GE Healthcare, Piscataway, NJ, USA). The band density was quantified using a computer-assisted imaging analysis system (ImageJ; U.S. Institutes of Health, Bethesda, MD, USA). Piezo2 protein expressions were normalized to the housekeeping gene GAPDH (1:10,000; Santa Cruz Biotechnology, Santa Cruz, CA, USA).

### 2.7. Chemicals

IL-1*β* was purchased from R&D systems (Minneapolis, MN, USA) and dissolved in Dulbecco's phosphate-buffered saline. D-GsMTx4 was purchased from Tocris (Bristol, UK) and dissolved in normal saline.

### 2.8. Experimental Protocols

#### 2.8.1. Effects of Blocking Piezo2 Pathway on Inflammatory Pain

Subcutaneously injected IL-1*β* (1 ng/50 *μ*L) into the vibrissa pad evoked significant mechanical allodynia and thermal hyperalgesia in the orofacial region (*n* = 7/group). D-GsMTx4 (10 *μ*g/10 *μ*L), a Piezo2 inhibitor, was subcutaneously administered 1 h after IL-1*β* (1 ng) injection (*n* = 7/group). Changes in air-puff thresholds and head withdrawal latency were measured at 1, 2, 3, 4, 5, 6, 7, 8, and 24 h after the subcutaneous injection of D-GsMTx4 or vehicle.

#### 2.8.2. Changes in Piezo2 Expression in the Trigeminal Ganglion and Trigeminal Subnucleus Caudalis

The present study investigated changes in Piezo2 expression in the ipsilateral trigeminal ganglion and trigeminal subnucleus caudalis in rats following an inferior alveolar nerve injury. Western blot analysis was conducted to evaluate changes in Piezo2 expression on POD 5 (*n* = 6/group).

#### 2.8.3. Effects of Blocking Piezo2 Pathway on Trigeminal Neuropathic Pain

An inferior alveolar nerve injury induced long-term nociceptive behavior in several previous studies [22, 23, 31, 38]. The present study also investigated mechanical allodynia resulting from an inferior alveolar nerve injury caused by improperly positioned dental implants. Changes in air-puff thresholds were measured at 1, 2, 3, 4, 7, 10, 14, 17, 21, 30, and 50 days after an inferior alveolar nerve injury (*n* = 7/group). On POD 5, D-GsMTx4 (5, 10 *μ*g/50 *μ*L) was administered subcutaneously in the vibrissa pad area. Changes in air-puff thresholds were measured at 1, 2, 4, 6, 8, 10, and 24 h after the injection of D-GsMTx4 or vehicle (*n* = 7/group). After administering D-GsMTx4 (5, 10 *μ*g/10 *μ*L) intracisternally, changes in air-puff thresholds were also examined at 1, 2, 4, 6, 8, 10, and 24 h (*n* = 7/group).

### 2.9. Data Analysis

Analysis of nociceptive behavior between groups involved repeated-measures analysis of variance followed by Holm–Sidak post hoc analysis. Analysis of Piezo2 expression changes utilized one-way ANOVA and Holm–Sidak post hoc analysis for multiple group comparisons. Student's *t*-test was used to compare the two groups. For all comparisons, a *p* value of less than 0.05 was considered to indicate statistical significance. All data were expressed as mean± standard error of the mean (SEM).

## 3. Results

### 3.1. Effect of Blocking Piezo2 Pathway on IL-1*β*–Induced Mechanical Allodynia and Thermal Hyperalgesia

**Figure 1:**
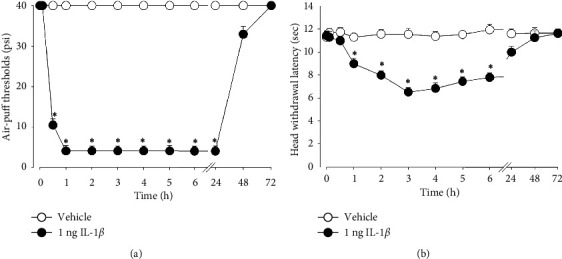
Interleukin (IL)-1*β*–induced mechanical allodynia and thermal hyperalgesia. (a) Subcutaneous injection of IL-1*β* (1 ng) significantly reduced the air-puff threshold compared to the vehicle group, resulting in mechanical allodynia for over 24 h. (b) Subcutaneous injection of IL-1*β* (1 ng) significantly reduced head withdrawal latency, leading to thermal hyperalgesia for up to 6 h. Vehicle administration does not affect either air-puff thresholds or head withdrawal latency. *n* = 7 animals per group. ^∗^*p* < 0.05. Vehicle- vs. IL-1*β*-treated group.

Figure 1 illustrates changes in air-puff thresholds and head withdrawal latency after the subcutaneous administration of IL-1*β* into the vibrissa pad area. The administration of the vehicle did not affect air-puff thresholds and head withdrawal latency. Subcutaneously injected IL-1*β* (1 ng) significantly attenuates air-puff thresholds (*F*_(1.2)_ = 0.619, *p* < 0.05, Figure 1(a)) and head withdrawal latency (*F*_(1.2)_ = 1.556, *p* < 0.05, Figure 1(b)). Mechanical allodynia induced by IL-1*β* was observed 30 min after injection, maintained until 24 h, and returned to the pretreated level at 48 h, in comparison with the group treated with the vehicle. Thermal hyperalgesia induced by IL-1*β* was also observed 1 h after injection and persisted for up to 6 h, in comparison with the vehicle-treated group.

**Figure 2:**
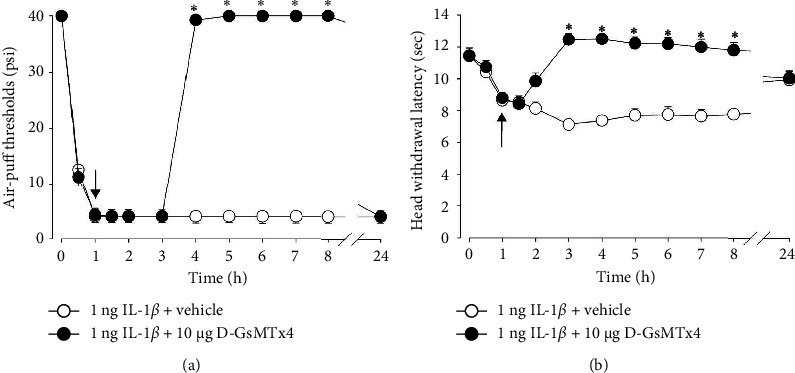
The effects of subcutaneously injected D-GsMTx4, a Piezo2 inhibitor, on IL-1*β*–induced pronociception. (a) Subcutaneous administration of D-GsMTx4 resulted in the inhibition of mechanical allodynia induced by the subcutaneous injection of IL-1*β* (1 ng). The antiallodynic effects were observed 4 h after the injection and persisted for up to 8 h. (b) Subcutaneous injection of D-GsMTx4 suppressed IL-1*β*–induced thermal hyperalgesia for up to 8 h. Vehicle administration does not affect the air-puff threshold or head withdrawal latency. *n* = 7 animals per group. ^∗^*p* < 0.05. Vehicle- vs. D-GsMTx4-treated group. The arrow indicates the injection of D-GsMTx4.

Effects of treatment with D-GsMTx4, a Piezo2 inhibitor, on IL-1*β*–induced mechanical allodynia and thermal hyperalgesia are illustrated in Figure 2. The Piezo2 inhibitor was administered into the subcutaneous tissue of the vibrissa pad area 1 h after IL-1*β* injection. Treatment with D-GsMTx4 (10 *μ*g) produced significant antiallodynic effects compared to vehicle treatment (*F*_(1.2)_ = 3.512, *p* < 0.05, Figure 2(a)). Subcutaneous injection of D-GsMTx4 increased air-puff thresholds 3 h after injection, and the antiallodynic effects were maintained until 7 h after injection. Treatment with D-GsMTx4 (10 *μ*g) also significantly inhibited thermal hyperalgesia compared to vehicle treatment (*F*_(1.2)_ = 0.903, *p* < 0.05, Figure 2(b)). Subcutaneous injection of D-GsMTx4 increased head withdrawal latency 3 h after injection, and the antinociceptive effects were maintained until 7 h after injection. The administration of the vehicle had no effects on either mechanical allodynia or thermal hyperalgesia.

### 3.2. Changes in Piezo2 Expression in the Trigeminal Ganglion and Trigeminal Subnucleus Caudalis Following Inferior Alveolar Nerve Injury

**Figure 3:**
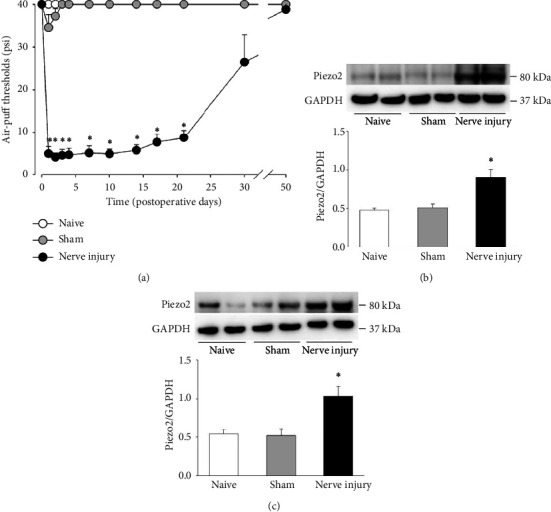
Changes in air-puff thresholds and Piezo2 expression following inferior alveolar nerve injury. (a) The misplacement of a dental implant resulted in significant mechanical allodynia in the experimental animals due to damage to the inferior alveolar nerve. Mechanical allodynia began to appear significantly from Day 1 after the nerve injury, lasted until 28 days postinjury, gradually recovered over time, and returned to the same level as before the nerve injury by Day 50. In the sham group, air-puff thresholds were not significantly altered compared to naïve rats. *n* = 7 animals per group. (b) and (c) Western blotting analysis showed upregulation of Piezo2 expression on POD 5 in the ipsilateral trigeminal ganglion (b) and trigeminal subnucleus caudalis (c). GAPDH was used as a loading control. *n* = 6 animals per group. ^∗^*p* < 0.05, comparing the sham group with the inferior alveolar nerve-injured group.

Figure 3 illustrates the changes in air-puff thresholds resulting from damage to the inferior alveolar nerve caused by improper placement of a dental implant, as well as changes in Piezo2 expression in the ipsilateral trigeminal ganglion and trigeminal subnucleus caudalis. Inferior alveolar nerve injury resulted in significant mechanical allodynia in the experimental animals (*F*_(1.2)_ = 33.447, *p* < 0.05, Figure 3(a)). Mechanical allodynia began to appear significantly from Day 1 after the nerve injury, persisted until 28 days postinjury, gradually improved over time, and returned to the preinjury level by Day 50. In the sham group, air-puff thresholds were not significantly altered compared to naïve rats.

Changes in Piezo2 expression in the trigeminal ganglion and trigeminal subnucleus caudalis following inferior alveolar nerve injury on POD 5 are illustrated in Figures 3(b) and 3(c). Western blot analysis revealed an upregulation of Piezo2 expression in the trigeminal ganglion and trigeminal subnucleus caudalis following inferior alveolar nerve injury compared to the naïve and sham groups (*p* < 0.05, Figures 3(b) and 3(c)).

### 3.3. Effects of Subcutaneously Injected Piezo2 Inhibitors on Neuropathic Mechanical Allodynia

**Figure 4:**
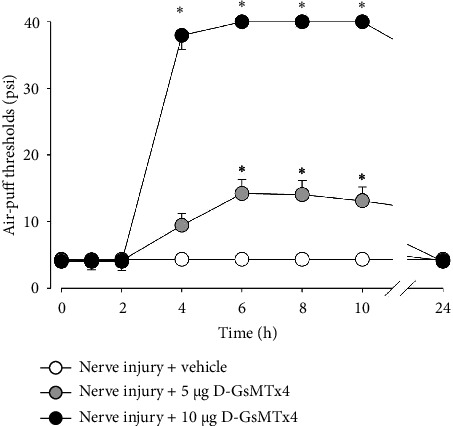
Effects of subcutaneous treatment with D-GsMTx4 on mechanical allodynia in rats with an inferior alveolar nerve injury. Subcutaneous administration of D-GsMTx4 (5 or 10 *μ*g), a Piezo2 inhibitor, produced antiallodynic effects 4 h after injection, which continued for up to 10 h. The vehicle administration did not affect the air-puff threshold. *n* = 7 animals per group. ^∗^*p* < 0.05. Vehicle- vs. D-GsMTx4-treated group.

Figure 4 illustrates changes in neuropathic mechanical allodynia and Piezo2 expression in the trigeminal ganglion following the subcutaneous administration of a Piezo2 inhibitor 5 days after an inferior alveolar nerve injury. In the control group, there were no significant effects on mechanical allodynia. Subcutaneous injection of 5 or 10 *μ*g of D-GsMTx4 significantly attenuated neuropathic mechanical allodynia compared to the vehicle-treated group (*F*_(2,18)_ = 18.708, *p* < 0.05, Figure 4). The antiallodynic effects were observed 4 h after the subcutaneous injection of 10 *μ*g of D-GsMTx4. The antiallodynic effects persisted for up to 10 h after the injection. The antiallodynic effects were restored 24 h after the drug injection.

### 3.4. Effect of Piezo2 Inhibitor Injected Intracisternally on Neuropathic Mechanical Allodynia

**Figure 5:**
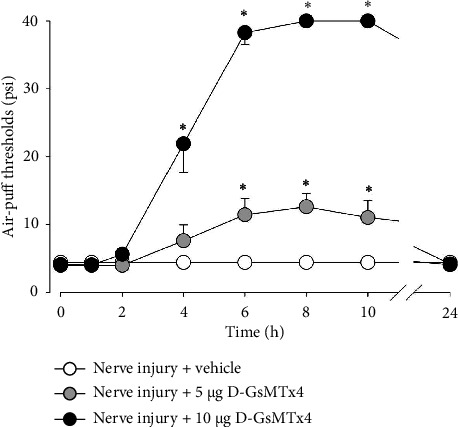
Effects of intracisternal treatment with D-GsMTx4 on mechanical allodynia in rats with an inferior alveolar nerve injury. Intracisternal administration of D-GsMTx4 (5 or 10 *μ*g), a Piezo2 inhibitor, produced antiallodynic effects 4 h after injection, which continued for up to 10 h. The vehicle administration did not affect the air-puff threshold. *n* = 7 animals per group. ^∗^*p* < 0.05. Vehicle- vs. D-GsMTx4-treated group.

Figure 5 illustrates the changes in neuropathic mechanical allodynia and Piezo2 expression in the trigeminal subnucleus caudalis when a Piezo2 inhibitor was administered intracisternally on POD 5. In the control group, the vehicle did not affect air-puff thresholds. Intracisternal injection of 5 or 10 *μ*g of D-GsMTx4 significantly attenuated neuropathic mechanical allodynia compared to the vehicle-treated group (*F*_(1.2)_ = 32.371, *p* < 0.05, Figure 5). The intracisternal injection of 10 *μ*g D-GsMTx4 produced antiallodynic effects 4 h after administration. The antiallodynic effects persisted for up to 10 h after injection, similar to peripheral actions. The antiallodynic effects were restored 24 h after the drug injection.

## 4. Discussion

The present study demonstrated that blocking the Piezo2 pathway attenuates nociceptive behavior in the orofacial area. Subcutaneous injection of a Piezo2 inhibitor specifically blocked IL-1*β*–induced mechanical allodynia and thermal hyperalgesia. Furthermore, subcutaneous and intracisternal injections of a Piezo2 inhibitor blocked neuropathic mechanical allodynia. Analysis of changes in Piezo2 expression also supported the behavioral evidence for antinociception resulting from blocking the Piezo2 pathway. Western blot analysis revealed an upregulation of Piezo2 expression in the trigeminal ganglion and the trigeminal subnucleus caudalis following inferior alveolar nerve injury. These results suggest that the Piezo2 pathway is involved in the development of inflammatory and neuropathic pain in the orofacial region. Blocking the Piezo2 pathway could serve as the foundation for developing new therapeutic strategies to address chronic orofacial pain conditions.

Generally, Piezo ion channels respond to shear stress caused by mechanical stimuli in various tissues [7–9]. Recently, emerging evidence has supported the involvement of Piezo2 in the processing of pain information. The expression of Piezo2 is localized in A*δ* axons of sensory neurons [13, 16], and Piezo2 expression was upregulated in the dorsal root ganglion and spinal cord dorsal horn neurons following CFA injection into the hind paw [18]. Moreover, injecting CFA into the knee joint resulted in knee swelling and joint pain, whereas the Piezo2 conditional knockout mouse did not exhibit knee swelling or joint pain in the experimental osteoarthritis animal model [39]. Mice lacking Piezo2 in sensory neurons also exhibited antinociceptive behavioral responses to mechanical stimuli, showing a reduced firing rate of A*δ*-nociceptor and C-fiber in response to mechanical stimulation [40]. These results suggest that Piezo2 is an essential component for detecting light touch or mechanical allodynia after injury in mice. These behavioral data are supported by experimental results showing complete desensitization to soft dynamic touch in Piezo2 knockout mice and humans [41]. The previous study also supported the involvement of Piezo2 in orofacial pain transmission. CFA injection induced Piezo2 expression in the trigeminal ganglion in control mice, while Piezo2 knockout mice blocked gentle mechanical stimulation in the orofacial area [41]. Therefore, Piezo2 might play a critical role in the development of various chronic pain conditions.

The present study used IL-1*β*–induced pronociception as an inflammatory pain model. Pain responses triggered by IL-1*β* have already been investigated in previous studies. Subcutaneously injected IL-1*β* resulted in pronociception such as mechanical allodynia and thermal hyperalgesia [21]. The present study also introduced that subcutaneously injected IL-1*β* produced pronociception, while vehicle administration did not. IL-1*β*–induced mechanical allodynia persisted for up to 24 h, while thermal hyperalgesia lasted for up to 6 h, compared to the vehicle treatment. Our results are consistent with those of previous studies on the importance of investigating inflammatory pain by assessing pain responses evoked by IL-1*β* injections. The present study also demonstrated that blocking the Piezo2 pathway by injecting a Piezo2 inhibitor into the vibrissa pad significantly inhibited mechanical allodynia and thermal hyperalgesia induced by subcutaneous injection of IL-1*β*. These results, taken together with previous studies, indicate that the peripheral Piezo2 pathway mediates inflammation-sensitized mechanical pain. Moreover, these results imply that targeting Piezo2 might be an effective strategy for treating inflammation-mediated hypersensitivity to tactile stimuli.

By blocking the Piezo2 pathway, we observed significant antinociceptive effects in rats with inferior alveolar nerve injury in this study. Subcutaneous injection of D-GsMTx4, a Piezo2 inhibitor, significantly attenuated neuropathic mechanical allodynia 4 h after injection compared to the vehicle-treated group. The antiallodynic effects sustained for up to 10 h after injection of D-GsMTx4. The results from western blot analysis provided additional support for the behavioral responses. Western blot analysis showed an upregulated Piezo2 expression in the trigeminal ganglion following an inferior alveolar nerve injury in comparison with the naïve and sham groups. The Piezo2 expression in the trigeminal ganglion was further supported by previous studies. Piezo2 expression was observed in the trigeminal ganglion following infraorbital nerve injury [20, 42]. Piezo2 expression was also observed in the axon, predominantly in the A*δ* axons that transmit nociceptive information, in rats and humans [16]. Moreover, intraperitoneal injection of D-GsMTx4 attenuated mechanical allodynia and hyperalgesia in rats with infraorbital nerve injury [20], and Piezo2 knockout mice did not exhibit mechanical allodynia [42]. Taken together, the present results indicate that the peripheral Piezo2 pathway influences neuropathic mechanical allodynia in the orofacial area.

The present study also investigated changes in neuropathic mechanical allodynia when a Piezo2 inhibitor was administered intracisternally on POD 5. The intracisternal injection of D-GsMTx4 significantly attenuated neuropathic mechanical allodynia compared to the vehicle-treated group. Moreover, western blot analysis supported the behavioral data by revealing an upregulation of Piezo2 expression in the trigeminal subnucleus caudalis following inferior alveolar nerve injury compared to the naïve and sham groups. These results suggest that the trigeminal Piezo2 pathway is involved in the development of neuropathic pain in the orofacial region following inferior alveolar nerve injury. Similarly, previous studies have reported the participation of the spinal Piezo2 pathway in the processing of nociceptive information. *In vitro* studies have also shown that the enhancement of Piezo2 current by mechanical stimulation of the sensory neurons is involved in mediating vincristine-induced mechanical hypersensitivity [43]. Intrathecal administration of Piezo2 antisense oligodeoxynucleotide attenuated Piezo2 expression in the spinal cord and mechanical allodynia in an EPAC1-dependent neuropathic animal model [44]. Moreover, intraperitoneal injection of D-GsMTx4, a Piezo2 inhibitor, inhibited mechanical allodynia and hyperalgesia induced by infraorbital nerve injury [20]. The present study also demonstrated that inferior alveolar nerve injury upregulated Piezo2 expression in the trigeminal spinal subnucleus caudalis and that blocking the Piezo2 pathway resulted in significant antiallodynic effects in the animals. These results further suggest that Piezo2 plays an important role in the development of neuropathic pain in the orofacial area.

The misplacement of a dental implant resulted in significant mechanical allodynia in the experimental animals due to damage to the inferior alveolar nerve. The present study utilized the alteration of air-puff thresholds after an inferior alveolar nerve injury induced by improperly positioned dental implants, as previously described [22, 23, 31]. The present study demonstrated that mechanical allodynia is observed 1 day after the nerve injury, persisted until 28 days postinjury, and returned to the preinjury level on POD 50. Therefore, the animal model used here showed pain behavior similar to symptoms reported in patients experiencing sensory changes due to alveolar nerve damage after dental implant surgery [45, 46]. These results suggest that the rat model of trigeminal neuropathic pain caused by malpositioned dental implants is also suitable for clinical research.

In the present study, we exclusively utilized male SD rats. This decision was made to minimize variability in responses, as research indicates that females generally exhibit lower pain thresholds and tolerance compared to males [47]. Sex hormones are believed to be a primary mechanism underlying the gender differences observed in pain processing [48]. Consequently, we focused solely on male SD rats to eliminate the influence of sex hormones on nociceptive thresholds. When conducting western blotting, only the ipsilateral dorsal region of the caudal medulla was used in the experiment. However, various surrounding structures, in addition to the medullary dorsal horn, such as the solitary tract nucleus, the hypoglossal (XII) nucleus, the vagus (X) nucleus, and the dorsal column nuclei, may be included in the final analysis. Consequently, these unavoidable variables may influence the western blot data. Our results suggest that mechanical neuropathic pain resulting from nerve damage is mediated by the Piezo2 receptor. Consequently, the Piezo2 receptor is likely to serve as a clinical target for pain management, and blocking the Piezo2 pathway may prove effective in treating patients with neuropathic pain. This hypothesis has already been partially supported by clinical trials. Szczot et al. reported that individuals with Piezo2 dysfunction exhibited higher thresholds for detecting pinch pain stimuli in hairy skin. However, the small sample size of only four patients limits the ability to draw definitive conclusions [49]. Therefore, further studies are necessary to elucidate the role of Piezo2 in pain management for patients. In a recent study, we observed that RIPK1 expression increased in the trigeminal nucleus caudalis following inferior alveolar nerve injury [50]. Specifically, the blockade of TNF-*α* produced antiallodynic effects and reduced RIPK1 expression. These results confirm that the TNF-*α*-mediated RIPK1 pathway is a potential therapeutic target for alleviating neuropathic pain after nerve injury. Furthermore, these findings suggest that additional substances may be involved alongside the Piezo pathway, indicating that further experiments are necessary to clarify this relationship.

In summary, the present study demonstrated that blocking the peripheral Piezo2 pathway attenuated IL-1*β*–induced mechanical allodynia and thermal hyperalgesia following subcutaneous injection. Moreover, neuropathic mechanical allodynia was blocked by both peripheral and spinal Piezo2 pathway blockade. In addition, inferior alveolar nerve injury significantly increased the expression of Piezo2 in the trigeminal ganglion and trigeminal subnucleus caudalis. These results demonstrate that the Piezo2 pathway plays a crucial role in the development of inflammatory and neuropathic pain in the orofacial region. Hence, blocking the Piezo2 pathway could serve as the basis for developing new therapeutic strategies to treat chronic orofacial pain conditions.

## Data Availability

The data used to support the findings of this study are available from the corresponding author upon reasonable request.
